# Admission monocyte-to-albumin ratio predicts 3-month functional outcomes after acute ischemic stroke: a retrospective cohort study

**DOI:** 10.3389/fneur.2025.1705544

**Published:** 2025-12-17

**Authors:** Huizhen Lu, Chuanliu Wang, Ming Yang, Yuanshao Lin, Weifeng Jiang

**Affiliations:** 1 Department of Neurology, The Quzhou Affiliated Hospital of Wenzhou Medical University (Quzhou People’s Hospital), Quzhou, Zhejiang, China; 2 Department of Neurology, The First Affiliated Hospital of Wenzhou Medical University, Wenzhou, China; 3 Department of Gerontology, The Quzhou Affiliated Hospital of Wenzhou Medical University, Quzhou People's Hospital, Quzhou, China

**Keywords:** acute ischemic stroke, monocyte-to-albumin ratio, inflammation, prognosis, risk stratification

## Abstract

**Background:**

The monocyte-to-albumin ratio (MAR) integrates systemic inflammation and nutritional status derived from routine laboratory data. We assessed whether the admission MAR is associated with 3-month functional outcomes following acute ischemic stroke (AIS).

**Methods:**

We conducted a single-center, retrospective cohort study of consecutive adults with AIS admitted within 3 days of symptom onset (October 2023–March 2024). MAR was calculated from the admission monocyte counts and serum albumin levels. The primary outcome was poor 3-month functional status, defined as a modified Rankin Scale (mRS) score ≥3. Associations between MAR and outcomes were examined using multivariable logistic regression (with and without adjustment), smooth curve fitting, and prespecified subgroup analyses (sex, age, smoking status, drinking status, hypertension, diabetes status, eGFR, and TOAST subtype).

**Results:**

Among 395 patients (mean age 66.2 years; 34.7% female), 59 (14.9%) had poor outcomes. A higher admission MAR independently predicted poor outcomes: per 1-unit increase, the adjusted odds ratio (OR) was 1.13 (95% CI 1.07–1.20; *p* < 0.001). Compared with the low tertile, patients with the high tertile had significantly greater odds (OR 3.21; 95% CI 1.25–8.20) with a linear trend (*P* for trend = 0.006). Smooth curve fitting demonstrated a largely monotonic increase in risk across the observed MAR range. Associations were consistent across subgroups with no significant interactions (all interactions *p* > 0.05). With respect to the TOAST subtype, the MAR remained significant for large-artery atherosclerosis (OR 1.10; 95% CI 1.02–1.20) and small-artery occlusion (OR 1.23; 95% CI 1.07–1.42), but not for cardioembolism.

**Conclusion:**

The admission MAR is independently and positively associated with poor 3-month functional outcomes after AIS. MAR is a promising tool for early risk assessment when it is integrated with established predictors.

## 1 Introduction

Acute ischemic stroke (AIS) remains a leading cause of death and long-term disability worldwide, imposing substantial individual and societal burdens through high mortality, recurrent events, and persistent functional impairment (1). With aging populations and persistent gaps in primary prevention, the global incidence and prevalence of AIS remain high, contributing to considerable years of life lost and disability-adjusted life years. Despite advances in reperfusion therapies and secondary prevention, early risk stratification to anticipate functional outcomes continues to be a clinical priority. Consequently, early and accurate risk stratification is a clinical imperative to guide acute management, optimize resource allocation, tailor secondary prevention strategies, and inform rehabilitation planning.

Accessible, inexpensive blood-based biomarkers that capture the host inflammatory response have attracted considerable interest in stroke prognostication (2, 3). Hematologic indices such as the remnant cholesterol inflammatory index are independent predictors of unfavorable 3-month outcomes (4). Among circulating leukocytes, monocytes are key effectors of innate immunity and inflammation after cerebral ischemia (5). These cells infiltrate ischemic tissues, amplify cytokine cascades, and contribute to tissue remodeling (5). Serum albumin appears to be neuroprotective during acute stroke, exerting multiple benefits including maintaining intravascular volume, exerting antioxidant and anti-inflammatory effects, and inhibiting platelet aggregation. Additionally, albumin serves as a marker of nutritional status and systemic inflammation, both of which are linked to adverse outcomes (6–8). Clinically, lower serum albumin levels predict worse functional outcomes and higher mortality after AIS (9).

Numerous studies have validated the prognostic utility of inflammation-based indices, such as the neutrophil-to-lymphocyte ratio (NLR), and nutrition-based indices, such as the prognostic nutritional index (PNI). However, these markers typically capture distinct aspects of the poststroke systemic response (10, 11). A biomarker that simultaneously integrates both the pro-inflammatory cellular response and the body’s nutritional and anti-inflammatory reserves could provide a more holistic prognostic assessment. The monocyte-to-albumin ratio (MAR) integrates these two complementary dimensions into a single metric calculated from routine tests. As a composite inflammatory index, the MAR has emerged as a prognostic marker across diverse conditions. A higher MAR independently predicted long-term mortality after percutaneous coronary intervention, underscoring its relevance in atherosclerotic cardiovascular disease (12). In acute brain injury, admission MAR was strongly associated with hematoma expansion after spontaneous intracerebral hemorrhage, a pathophysiological process similarly driven by early inflammatory responses (13). Beyond the cardiovascular and neurovascular fields, the MAR has also been linked to outcomes in patients with chronic liver disease, supporting its role as a general marker of systemic inflammation (14). Despite these advances, whether the MAR measured at admission is associated with functional prognosis after AIS still remains to be established.

Given the central role of monocyte-mediated inflammation in ischemic brain injury and the protective, anti-inflammatory functions of albumin, we hypothesized that a higher MAR would be related to worse 3-month functional outcomes after AIS. Accordingly, we investigated whether the admission MAR is associated with 3-month functional outcomes in AIS patients, with the aim of determining its incremental prognostic value and assessing its utility in identifying patients at increased risk of poor recovery.

## 2 Methods

### 2.1 Study design

This study is a single-center retrospective cohort study of consecutive adult AIS patients admitted from October 2023 to March 2024. The protocol complied with the Declaration of Helsinki and was approved by the Institutional Review Board. Owing to the retrospective design and de-identified data, the requirement for informed consent was waived.

### 2.2 Study population

The inclusion criteria were: age ≥18 years; AIS was identified using the International Classification of Diseases, Ninth Revision, Clinical Modification (ICD-9-CM) codes 433 and 434, and the International Classification of Diseases, Tenth Revision, Clinical Modification (ICD-10-CM) code I63 and confirmed by neuroimaging (CT or MRI) that excluded hemorrhage and identified an acute ischemic lesion consistent with the clinical presentation; admission within 3 days of symptom onset; availability of admission laboratory tests including monocyte count and serum albumin; documented National Institutes of Health Stroke Scale (NIHSS) score at admission; and documented the modified Rankin scale (mRS) score at admission and at 3 months. The exclusion criteria included active infection, hematologic disorders, hepatic insufficiency, and receipt of intravenous thrombolysis or mechanical thrombectomy.

### 2.3 Measurement of MAR

Peripheral venous blood samples were collected at admission as part of routine clinical care, generally within 24 h. The monocyte count was measured at 10^9/L using an automated hematology analyzer, and serum albumin concentration was measured in g/L using standardized biochemical methods in the hospital’s central laboratory. MAR was calculated as MAR = the ratio of monocyte count × 1,000 to the albumin level.

### 2.4 Outcomes

Functional status at 3 months post-stroke was evaluated using the mRS by trained personnel, either during an outpatient visit or through a standardized telephone interview. A poor outcome was defined as an mRS score ≥3 (i.e., 3–6), whereas a good outcome was defined as an mRS score of 0–2.

### 2.5 Covariates

Covariate selection was guided by clinical relevance and prior literature on AIS prognosis. The following variables were considered as potential confounders and/or effect modifiers and were extracted from the medical records at baseline unless stated otherwise: Demographics/behaviors: age, sex, current smoking status, and current drinking status. Vascular risk factors/comorbidities: diabetes mellitus, hypertension, and atrial fibrillation. Stroke characteristics: baseline NIHSS score; ischemic stroke etiology per TOAST classification, including large-artery atherosclerosis (LAA), cardioembolism (CE), small-artery occlusion (SAO), stroke of other determined etiology (SOE), and stroke of undetermined etiology (SUE). Renal function and lipid profile: estimated glomerular filtration rate (eGFR), triglyceride (TG), total cholesterol (TC), low-density lipoprotein cholesterol (LDL-C), and high-density lipoprotein cholesterol (HDL-C) levels. Additional descriptive laboratory indices included white/red blood cell counts, platelet count, hemoglobin, globulin, alanine aminotransferase (ALT), aspartate aminotransferase (AST), blood urea nitrogen, creatinine, fasting blood glucose, and C-reactive protein (CRP).

### 2.6 Statistical analysis

Baseline features were summarized overall and across MAR tertiles. Depending on distribution, continuous variables are expressed as the mean ± SD or median (min–max) and were compared via one-way ANOVA or the Kruskal–Wallis test. Categorical data are summarized as counts (percentages) and were compared using χ^2^ tests. The amount of missing data for the variables included in the final regression models was minimal (<5%); therefore, any patient records with missing values for these variables were excluded from the analysis.

Associations between candidate variables and poor 3-month outcomes were first examined using univariate logistic regression. The association between the MAR and poor 3-month outcomes was assessed using logistic regression under three prespecified models: Model 1: unadjusted; Model 2: adjusted for age, sex, smoking status, and alcohol consumption status; Model 3: further adjusted for hypertension, diabetes mellitus, TOAST subtype, eGFR, baseline NIHSS, and LDL-C. The MAR was analyzed as a continuous predictor (per 1-unit increase) and by tertiles (low as reference). A linear trend across tertiles was evaluated by assigning each tertile to its median and entering that value as a continuous variable. To explore potential nonlinearity between continuous MAR and the probability of poor outcome, we fitted a smoothed curve using restricted cubic splines within Model 3. Prespecified stratified analyses examined the consistency of associations across strata of sex, age group (≤65 vs. >65 years), smoking status, alcohol consumption status, hypertension status, diabetes status, TOAST subtype, and renal function (eGFR).

## 3 Results

### 3.1 Baseline characteristics by MAR tertiles

In the final analytic sample of 395 patients with AIS, 336 (85.06%) had favorable 3-month outcomes (mRS ≤ 2), while 59 (14.94%) had unfavorable outcomes (mRS > 2). Patients were stratified by the admission MAR tertiles: Low, Middle, and High. The mean age was 66.20 ± 11.93 years, and the difference was not significant (*p* = 0.067). The proportion of female patients decreased across the MAR tertiles (51.9, 34.1, and 18.2%; *p* < 0.001). Smoking status and drinking status were more frequent in the higher MAR groups (*p* = 0.049). The prevalence of diabetes mellitus and hypertension was similar among the tertiles (Table 1).

**Table 1:** Baseline characteristics of acute ischemic stroke patients stratified by admission monocyte-to-albumin ratio tertiles (*n* = 395).

Characteristics	Overall (*n* = 395)	Low (<10.0) (*n* = 131)	Middle (10.0–14.9) (*n* = 132)	High (>14.9) (*n* = 132)	*p*-value
Age, years	66.20 ± 11.93	65.34 ± 11.93	65.08 ± 11.64	68.16 ± 12.07	0.067
Female, *n* (%)	137 (34.7)	68 (51.9)	45 (34.1)	24 (18.2)	<0.001
Current smoking status, *n* (%)	174 (44.1)	40 (30.5)	59 (44.7)	75 (56.8)	<0.001
Current drinking status, *n* (%)	163 (41.3)	43 (32.8)	58 (43.9)	62 (47.0)	0.049
Hypertension, *n* (%)	313 (79.2)	103 (78.6)	107 (81.1)	103 (78.0)	0.813
Diabetes mellitus, *n* (%)	135 (34.2)	44 (33.6)	47 (35.6)	44 (33.3)	0.913
WBC, ×10^9^/L	7.41 ± 2.47	6.27 ± 2.18	7.39 ± 1.98	8.55 ± 2.65	<0.001
Monocytes, ×10^9^/L	0.49 ± 0.20	0.31 ± 0.06	0.46 ± 0.06	0.70 ± 0.19	<0.001
RBC, ×10^12^/L	4.48 ± 0.58	4.42 ± 0.54	4.54 ± 0.58	4.46 ± 0.61	0.232
Hemoglobin, g/L	136.15 ± 16.24	133.84 ± 15.05	138.03 ± 16.87	136.57 ± 16.57	0.105
Platelets, ×10^9^/L	231.35 ± 60.58	226.12 ± 58.59	233.74 ± 57.43	234.14 ± 65.51	0.482
Albumin, g/L	37.48 ± 3.24	38.30 ± 3.01	38.15 ± 2.93	36.00 ± 3.26	<0.001
Globulin, g/L	28.95 ± 4.05	28.73 ± 3.89	28.53 ± 3.71	29.60 ± 4.46	0.075
ALT, U/L	20.89 ± 18.24	23.43 ± 25.07	20.33 ± 11.59	18.94 ± 15.24	0.124
AST, U/L	24.31 ± 14.64	26.32 ± 21.06	23.19 ± 9.19	23.43 ± 10.66	0.155
Fasting blood glucose, mmol/L	6.77 ± 2.93	6.94 ± 2.97	6.66 ± 2.74	6.72 ± 3.09	0.721
Blood urea nitrogen, mmol/L	5.40 ± 1.80	5.32 ± 1.54	5.33 ± 1.75	5.56 ± 2.08	0.479
Creatinine, μmol/L	75.13 ± 23.22	70.65 ± 20.45	76.67 ± 23.41	78.05 ± 25.04	0.023
eGFR, mL/min/1.73 m^2^	88.65 ± 20.41	89.79 ± 19.67	87.63 ± 19.68	88.54 ± 21.91	0.691
Total cholesterol, mmol/L	4.67 ± 1.12	4.74 ± 1.20	4.79 ± 1.05	4.47 ± 1.09	0.042
Triglycerides, mmol/L	1.70 ± 1.14	1.73 ± 1.50	1.83 ± 0.98	1.53 ± 0.83	0.086
HDL-C, mmol/L	1.05 ± 0.26	1.09 ± 0.27	1.05 ± 0.27	1.01 ± 0.24	0.053
LDL-C, mmol/L	2.69 ± 0.90	2.73 ± 0.95	2.80 ± 0.86	2.55 ± 0.87	0.069
Homocysteine, μmol/L	14.01 ± 7.57	12.96 ± 5.39	14.21 ± 6.84	14.86 ± 9.74	0.118
CRP, mg/L	5.37 ± 6.99	3.66 ± 4.87	3.91 ± 4.33	8.53 ± 9.47	<0.001
Atrial fibrillation, *n* (%)	50 (12.7)	13 (9.9)	13 (9.8)	24 (18.2)	0.065
TOAST subtype, *n* (%)					0.038
LAA	203 (51.4)	56 (42.7)	75 (56.8)	72 (54.5)	
CE	57 (14.4)	19 (14.5)	13 (9.8)	25 (18.9)	
SAO	134 (33.9)	55 (42.0)	44 (33.3)	35 (26.5)	
SOE	1 (0.3)	1 (0.8)	0 (0.0)	0 (0.0)	
SUE	0 (0.0)	0 (0.0)	0 (0.0)	0 (0.0)	
Baseline NIHSS	4.27 ± 3.86	3.81 ± 2.83	3.77 ± 2.86	5.22 ± 5.22	0.002
Baseline mRS, *n* (%)					0.022
1	62 (15.7)	20 (15.3)	26 (19.7)	16 (12.1)	
2	209 (52.9)	77 (58.7)	66 (50.0)	66 (50.0)	
3	65 (16.5)	17 (13.0)	27 (20.5)	21 (15.9)	
4	42 (10.6)	14 (10.7)	11 (8.3)	17 (12.9)	
5	17 (4.3)	3 (2.3)	2 (1.5)	12 (9.1)	

Values are mean ± SD or *n* (%). *p*-values from one-way ANOVA (continuous) or χ^2^ test (categorical). AIS, acute ischemic stroke; MAR, monocyte-to-albumin ratio; WBC, white blood cells; RBC, red blood cells; ALT, alanine aminotransferase; AST, aspartate aminotransferase; eGFR, estimated glomerular filtration rate; HDL-C, high-density lipoprotein cholesterol; LDL-C, low-density lipoprotein cholesterol; CRP, C-reactive protein. NIHSS, National Institutes of Health Stroke Scale; mRS, modified Rankin Scale. TOAST, Trial of ORG 10172 in Acute Stroke Treatment, including large-artery atherosclerosis (LAA), cardioembolism (CE), small-vessel occlusion (SAO), stroke of other determined etiology (SOE), and stroke of undetermined etiology (SUE).

WBC and monocyte counts increased from the Low to High tertiles (both *p* < 0.001), whereas the serum ALB concentration was lower in the High group (*p* < 0.001). CRP was highest in the High tertile (*p* < 0.001). Renal indices were higher in the High group (*p* = 0.023), with comparable eGFRs and BUN levels across groups (*p* = 0.691 and *p* = 0.479), respectively. With respect to lipids, the TC level was lower in the High tertile (*p* = 0.042), while the HDL-C and LDL-C levels were not significantly different (*p* = 0.053 and *p* = 0.069). The full details are shown in Table 1.

### 3.2 Univariate predictors of poor 3-month outcomes

Based on univariable logistic regression (Table 2), older age was correlated with higher odds of poor outcomes (OR 1.07, 95% CI 1.04–1.10; *p* < 0.001). Compared with LAA, CE was linked to increased odds (OR 1.97, 95% CI 1.01–3.86; *p* = 0.048), whereas SAO was associated with lower odds (OR 0.22, 95% CI 0.09–0.53; *p* < 0.001). Low serum albumin levels (OR 0.89, 95% CI 0.82–0.97; *p* = 0.010) and high monocyte counts (OR 32.42, 8.45–124.43; *p* < 0.001) were significantly related to poor 3-month outcome. High BUN levels (OR 1.20, 95% CI 1.04–1.37; *p* = 0.010) and low eGFRs (OR 0.98, 95% CI 0.97–0.99; *p* = 0.001) were also related to poor outcomes. Baseline NIHSS score was strongly associated (OR 1.57, 95% CI 1.40–1.77; *p* < 0.001). Sex, smoking status, drinking status, hypertension status, diabetes status, LDL-C level, and fasting glucose level were not significantly associated with poor outcomes according to univariate analyses (all *p* > 0.05).

**Table 2:** Univariate predictors of poor 3-month functional outcome (modified Rankin Scale, mRS ≥ 3).

Variables	Mean ± SD/N (%)	OR (95%CI)	*P*-value
Sex			
Female	137 (34.68%)	Reference	
Male	258 (65.32%)	1.67 (0.89, 3.13)	0.108
Age (years)	66.20 ± 11.93	1.07 (1.04, 1.10)	<0.001
Smoking status			
No	221 (55.95%)	Reference	
Yes	174 (44.05%)	1.18 (0.68, 2.05)	0.568
Drinking status			
No	232 (58.73%)	Reference	
Yes	163 (41.27%)	0.89 (0.51, 1.58)	0.699
Hypertension			
No	82 (20.76%)	Reference	
Yes	313 (79.24%)	1.03 (0.52, 2.05)	0.931
Diabetes mellitus			
No	260 (65.82%)	Reference	
Yes	135 (34.18%)	1.51 (0.86, 2.66)	0.152
Albumin, g/L	37.48 ± 3.24	0.89 (0.82, 0.97)	0.010
Monocyte count, ×10^9^/L	0.49 ± 0.20	32.42 (8.45, 124.43)	<0.001
Fasting blood glucose, mmol/L	6.77 ± 2.93	1.05 (0.96, 1.14)	0.294
Blood urea nitrogen, mmol/L	5.40 ± 1.80	1.20 (1.04, 1.37)	0.010
eGFR, mL/min/1.73 m^2^	88.65 ± 20.41	0.98 (0.97, 0.99)	0.001
LDL C, mmol/L	2.69 ± 0.90	1.02 (0.75, 1.38)	0.921
Baseline NIHSS points	4.27 ± 3.86	1.57 (1.40, 1.77)	<0.001
Baseline mRS			
1	62 (15.70%)	Reference	
2	209 (52.91%)	1.51 (0.32, 7.07)	0.603
3	65 (16.46%)	6.11 (1.30, 28.82)	0.022
4	42 (10.63%)	30.00 (6.48, 138.99)	<0.001
5	17 (4.30%)	225.00 (29.26, 1730.44)	<0.001
TOAST subtype			
LAA	203 (51.39%)	Reference	
CE	57 (14.43%)	1.97 (1.01, 3.86)	0.048
SAO	134 (33.92%)	0.22 (0.09, 0.53)	<0.001
SOE	1 (0.25%)	0.00 (0.00, Inf)	0.988

### 3.3 Associations between the MAR and the 3-month functional outcomes

The relationship between the admission MAR and poor 3-month functional outcomes (mRS ≥ 3) was evaluated using multivariable logistic regression. Higher MAR was significantly related to increased odds of poor outcomes across all models: per 1-unit increase, the ORs were 1.14 (95% CI 1.09–1.19; *p* < 0.001) in Model 1, 1.13 (95% CI 1.07–1.18; *p* < 0.001) following adjustment for age, sex, smoking status, and alcohol consumption status (Model 2), and 1.13 (95% CI 1.07–1.20; *p* < 0.001) with further adjustment for hypertension, diabetes, TOAST subtype, eGFR, NIHSS, and LDL-C (Model 3). Notably, when modeled by tertiles, the High MAR group had significantly greater odds of poor outcomes than the Low group did in Models 2 and 3, with a linear dose–response pattern that remained significant after full adjustment (*P* for trend = 0.006) (Table 3).

**Table 3:** Multivariable logistic regression analysis of the association between monocyte-to-albumin ratio (MAR) and poor 3-month functional outcome.

	Model 1		Model 2		Model 3	
	OR (95%CI)	*P*-value	OR (95%CI)	*P*-value	OR (95%CI)	*P*-value
MAR	1.14 (1.09, 1.19)	<0.001	1.13 (1.07, 1.18)	<0.001	1.13 (1.07, 1.20)	<0.001
MAR tertile						
Low	Reference		Reference		Reference	
Middle	0.73 (0.30, 1.79)	0.485	0.71 (0.28, 1.78)	0.463	0.70 (0.23, 2.19)	0.543
High	4.01 (1.99, 8.10)	<0.001	3.30 (1.56, 6.97)	0.002	3.21 (1.25, 8.20)	0.015
*P* for trend		<0.001		<0.001		0.006

Model 1: Unadjusted.

Model 2: Adjusted for age, sex, smoking status, and drinking status.

Model 3: Further adjusted for hypertension, DM, TOST, eGFR, NIHSS, and LDL-C.

### 3.4 Subgroup and interaction analyses

**Figure 1:**
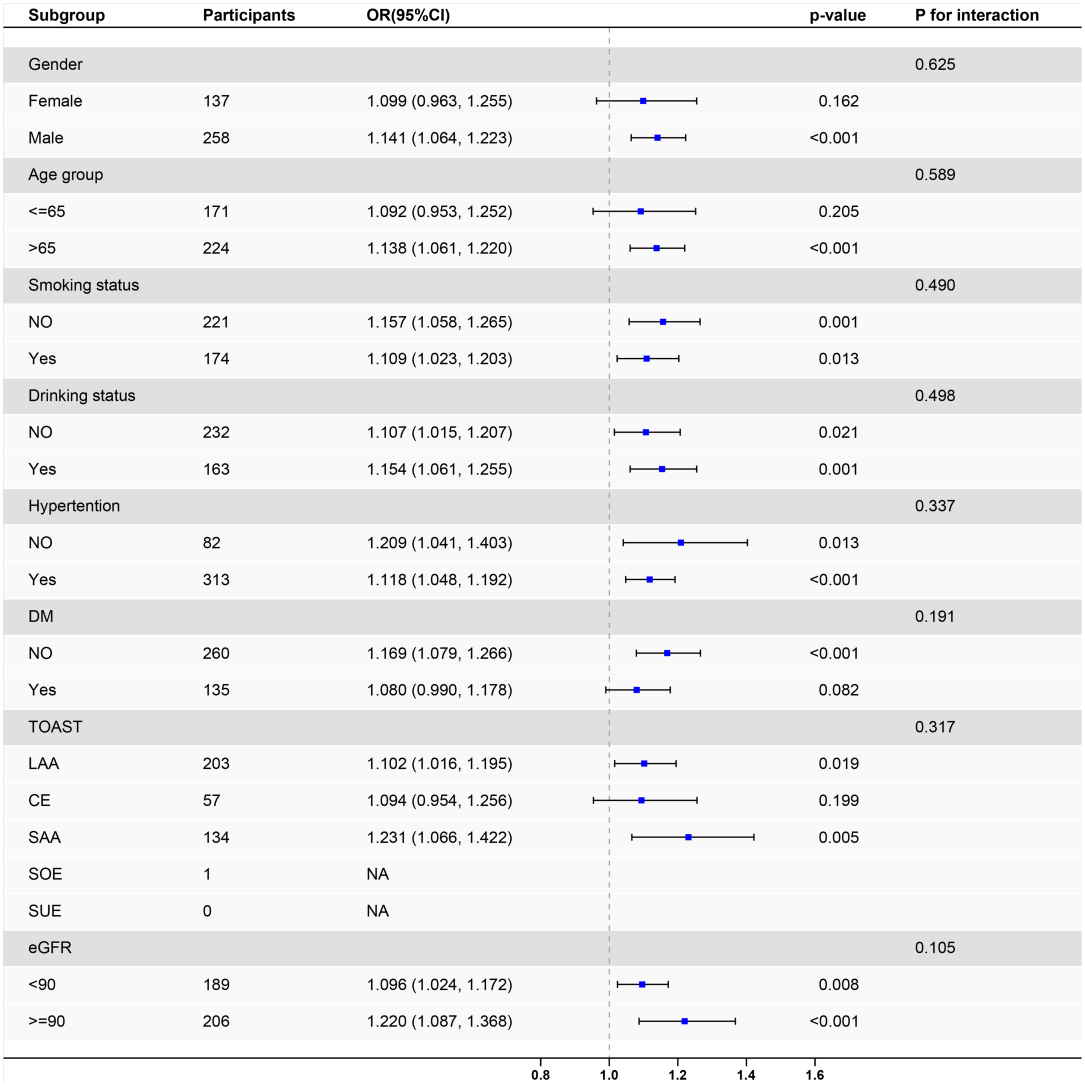
Forest plot depicting the association between monocyte-to-albumin ratio and 3-month modified Rankin Scale across clinical subgroups.

In stratified analyses, the positive association between the admission MAR and poor 3-month outcomes persisted across the subgroups, and was independent of smoking status, alcohol consumption status, hypertension status, diabetes status, and renal function. Significant associations were observed in men and in participants aged >65 years. With respect to the TOAST subtype, the association remained significant for the large-artery atherosclerosis (LAA) subtype and small-artery occlusion (SAO) subtype, whereas that for the cardioembolism (CE) subtype did not reach significance. The stroke of other determined etiology (SOE) and stroke of undetermined etiology (SUE) subtypes were not evaluated because few or no cases were included. No significant interactions were detected for sex, age group, smoking status, alcohol consumption status, hypertension status, diabetes status, TOAST subtype, or eGFR (*P* for interaction >0.05), indicating that the associations were consistent across subgroups (Figure 1).

### 3.5 Dose–response relationship between the MAR and poor 3-month functional outcomes

**Figure 2:**
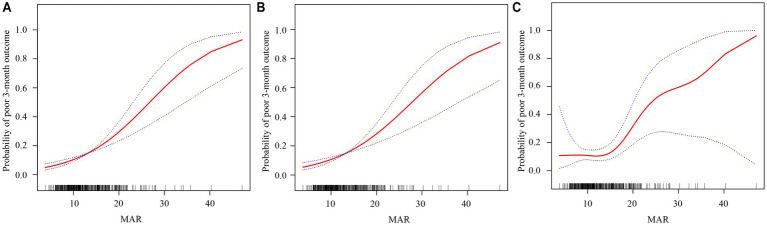
Dose–response relationships between the monocyte-to-albumin ratio (MAR) and a poor 3‑month functional outcome. The solid red line represents the smooth curve fit, and the blue bands indicate the 95% confidence interval. Three statistical models are presented: **(A)** Model 1 is the unadjusted analysis; **(B)** Model 2 is adjusted for age, sex, smoking status, and drinking status; and **(C)** Model 3 is further adjusted for hypertension, DM, TOST, eGFR, NIHSS, and LDL-C.

Afterward, smoothed curve fitting was used to determine the dose–response relationships between continuous MAR and the probability of poor 3-month outcomes across the three adjustment schemes (Figures 2A–C), indicating that the risk increased with increasing MAR over the observed range.

To further explore the dose–response relationship between the MAR and poor functional outcomes, a threshold effect analysis was performed using a piecewise linear regression model (Table 4). Below this threshold (MAR = 13.75), no significant association was observed between the MAR and poor outcomes (OR 0.97, 95% CI 0.81–1.17, *p* = 0.765). In contrast, above the inflection point, the association became markedly stronger, with each unit increase in MAR conferring a 19.4% increase in the odds of poor outcome (OR 1.19, 95% CI 1.09–1.31, *p* < 0.001). These findings suggest that the prognostic value of the MAR is most pronounced when values exceed approximately 13.75.

**Table 4:** Threshold effect analysis of mar on poor 3-month functional outcome.

Outcome	*β* (95%CI)	*p*-value
Model I		
Standard linear model	1.13 (1.07, 1.20)	<0.001
Model II		
Inflection point (K)	13.75	
<K	0.97 (0.81, 1.17)	0.765
>K	1.19 (1.09, 1.31)	<0.001

K, inflection point; Adjusted for age, sex, smoking status, drinking status, hypertension, DM, TOST, eGFR, NIHSS, and LDL-C.

## 4 Discussion

In this consecutive AIS cohort, a higher admission MAR independently predicted a greater risk of poor 3-month functional outcome, and the association persisted after stepwise adjustment for demographics, lifestyle, comorbidities, renal function, baseline stroke severity, and lipid profile. Smoothing analyses suggested a monotonic, dose–response relationship between the MAR and unfavorable outcome risk. Subgroup analyses stratified by smoking, drinking, hypertension, and eGFR yielded broadly consistent results, and interaction tests indicated that the association did not differ significantly by sex, age, diabetes mellitus, or TOAST subtype. Together, these findings support the notion that the MAR represents a stable correlate of poststroke functional prognosis.

Inflammation is a key determinant of prognosis after AIS, shaping both early tissue injury and subsequent recovery (15, 16). Within minutes of arterial occlusion, damage-associated molecular patterns activate microglia and endothelial cells, triggering the release of pro-inflammatory cytokines (e.g., TNF-*α* and IL-6) and adhesion molecules that recruit peripheral leukocytes, notably neutrophils and monocytes (17). These effector cells generate reactive oxygen species and proteases, disrupt the blood–brain barrier, drive vasogenic edema, and increase the risk of hemorrhagic transformation (18). Nutrition is likewise a pivotal, yet often under-recognized, determinant of outcome after AIS (19). Pre-existing or early poststroke malnutrition compromises immune competence, delays tissue repair, and undermines neuroplasticity, thereby leading to complications (e.g., infections and pressure injuries) and poor functional recovery (20–22). Among circulating markers, serum albumin is a practical proxy for host nutritional and antioxidant reserves as well as capillary integrity (23). Albumin, synthesized in the liver, serves not only as a marker of nutritional status but also as an indicator of systemic inflammation and oxidative stress (24).

According to the above findings, the MAR, a ratio of monocyte and albumin that integrates inflammation and nutritional status, may be a potential predictor of functional prognosis after AIS. However, no relevant studies have explored the ability of the MAR to predict functional prognosis after AIS, leaving a major knowledge gap. In this study of 395 patients, multivariable models consistently demonstrated that higher admission MAR was independently associated with a greater likelihood of poor 3-month functional outcome, even after adjustment for demographics, vascular risk factors, stroke etiology, renal function, baseline severity, and lipid parameters. The stability of the association across progressively adjusted models and alternative parameterizations (continuous and categorical) suggests that the signal is not an artifact of confounding or model specification. Clinically, the per-unit effect appears modest, but the coherence of findings across models implies potential utility as part of a composite risk profile rather than a stand-alone predictor.

Our findings, which establish the MAR as a prognostic marker, align with the growing body of evidence highlighting the pivotal role of inflammation in the pathophysiology of ischemic stroke. Systemic inflammation contributes to endothelial dysfunction and arterial stiffness, both of which are key processes in the development and progression of cerebrovascular disease (25). This inflammatory state is often intertwined with metabolic comorbidities, creating a synergistic effect that can worsen vascular health and neurocognitive function (26, 27). Such a pro-inflammatory environment not only increases the risk of the initial ischemic event but may also amplify the secondary inflammatory cascade following stroke, thereby contributing to greater stroke severity and poorer functional outcomes. Indeed, the impact of inflammation on vascular health is common in various systemic conditions and has been linked to immune response genes, further underscoring its fundamental role in stroke susceptibility and pathogenesis (28, 29). Moreover, elevated monocyte counts reflect systemic immune activation, and circulating monocytes can infiltrate the ischemic brain parenchyma, where they interact with resident microglia to amplify neuroinflammatory cascades (30). Conversely, in addition to its nutritional role, albumin exerts multiple neuroprotective effects through several nononcotic mechanisms, including the molecular transportation and free radical scavenging, the modulation of capillary permeability, the regulation of neutrophil adhesion and activation, and hemostatic effects (31). Low albumin levels may therefore impair these protective mechanisms, rendering the brain more vulnerable to inflammatory injury. Therefore, the elevated MAR observed in patients with poor outcomes in our cohort may represent a confluence of both a preexisting inflammatory burden and an acute-phase reaction to the cerebral injury. Subgroup findings reinforce the generalizability of the association between MAR and outcome across common clinical strata. The effects were broadly consistent regardless of smoking status, alcohol use, hypertension, diabetes, or renal function, and interaction testing did not identify any meaningful effect modification. The stronger associations in men and in older adults are plausibly explained by differences related to sex and age. Sex differences in immune responses, partly driven by sex hormones and genetics, also shape monocyte phenotypes and downstream inflammatory signaling (32, 33). Aging is characterized by “inflammaging,” with chronically elevated cytokine and pro-inflammatory monocyte activity (34, 35). Older adults often have a higher baseline pro-inflammatory state (34). From an etiological perspective, in LAA and SAO, inflammation and microvascular injury are central to disease mechanisms. Since the MAR captures systemic inflammatory activity through monocytes and the overall inflammatory–nutritional balance through albumin, it is biologically aligned with these pathways and thus more likely to be correlated with prognosis. In contrast, cardioembolic stroke is driven primarily by emboli originating in the heart, often due to atrial fibrillation and atrial pathology. As a result, the association of the MAR with outcomes in patients with cardioembolic stroke may be weak or inconsistent.

An intriguing finding of our study is that the prognostic value of MAR was significant in patients with LAA and SAO, but not in those with CE. This differential association is pathophysiologically plausible. Atherosclerosis, the underlying cause of LAA and often implicated in SAO (microatheroma), is a chronic inflammatory disease in which monocytes play a critical role (36). Monocytes infiltrate the arterial wall, differentiate into macrophages, and contribute to all stages of plaque development, from initiation to rupture. Therefore, a higher MAR in these patients likely reflects a greater systemic inflammatory burden and a more unstable atherosclerotic process, which directly translates to a more severe stroke and poorer outcome. In contrast, the primary driver of a cardioembolic stroke is the dislodgment of a preformed thrombus from a cardiac source (e.g., in atrial fibrillation). While inflammation is a consequence of the resulting cerebral ischemia, the initial event is less dependent on the patient’s systemic inflammatory state as measured by admission MAR. The clinical outcomes in CE may be more strongly influenced by factors such as embolus size, collateral circulation patency, and underlying cardiac function, rather than peripheral inflammatory markers. Our findings therefore suggest that the utility of the MAR as a prognostic tool may be most pronounced in stroke etiologies with a strong, direct inflammatory component.

## 5 Strengths and limitations

This study utilized a consecutive, real-world cohort, thereby enhancing clinical relevance and reducing selection bias. Rigorous adjustment was performed for potential confounders, including demographics, vascular comorbidities, renal function, lipid profile, and baseline NIHSS score. In addition, a generalized additive mixed model was employed to graphically depict the dose–response relationship between the MAR and functional outcomes. The results were consistent across clinically relevant subgroups, supporting the robustness and generalizability of the study population. Because the MAR is derived from inexpensive, standardized admission laboratory tests, it is readily implementable across care settings and suitable for rapid bedside risk stratification.

Nonetheless, important limitations should be acknowledged. First, the single-center, retrospective design raises the possibility of selection bias and residual confounding. Second, reliance on a single admission measurement of the MAR prevents the assessment of temporal dynamics and trajectory-based risk; unmeasured influences on monocytes and albumin, such as intercurrent infection, hydration status, and hepatic function, cannot be fully excluded. Third, the relatively low number of patients with poor functional outcomes is a limitation, which may impact the statistical robustness of our multivariable models and the generalizability of our findings. Fourth, although the multivariable models included major vascular and demographic factors, prior stroke history, nutritional supplementation, and in-hospital infections, all of which can influence albumin levels and outcomes, they were not explored in this study and warrant investigation in future research. Finally, the exclusion of patients with hepatic dysfunction, active infections, hematologic diseases, or those receiving acute reperfusion therapies (intravenous thrombolysis or mechanical thrombectomy) limits the generalizability of our findings to the broader acute ischemic stroke population. Future studies should investigate whether the prognostic value of the MAR extends to patients undergoing these interventions, clarifying its utility across a broader, higher-risk patient population. Prospective, multicenter validation should also be prioritized to establish the generalizability and clinical transportability of MAR-based risk stratification.

## 6 Conclusion

Admission MAR is independently and positively associated with poor 3-month functional outcomes after AIS, demonstrating a largely monotonic dose–response relationship and consistent effects across key clinical subgroups. Given its simplicity and widespread availability, MAR shows promise for early risk assessment and stratified management. However, given the single-center retrospective design of this study, these findings should be considered as preliminary. Large-scale, multicenter prospective validation studies are urgently needed to validate the reproducibility and clinical utility of MAR before it can be recommended for routine clinical application.

## Data Availability

The raw data supporting the conclusions of this article will be made available by the authors, without undue reservation.

## References

[1] GBD 2021 Stroke Risk Factor Collaborators. Global, regional, and national burden of stroke and its risk factors, 1990-2021: a systematic analysis for the global burden of disease study 2021. Lancet Neurol. (2024) 23:973–1003. doi: 10.1016/s1474-4422(24)00369-7, 39304265 PMC12254192

[2] Zhang L Antabi MA Mattar J Bounkari OE Fang R Waegemann K . Circulating cytokine levels and 5-year vascular recurrence after stroke: a multicenter prospective cohort study. Eur Stroke J. (2025):23969873251360145. doi: 10.1177/23969873251360145 40790506 PMC12343552

[3] Ma L Ji L Cheng Z Geng X Ding Y. Developing an explainable prognostic model for acute ischemic stroke: combining clinical and inflammatory biomarkers with machine learning. Brain Behav. (2025) 15:e70673. doi: 10.1002/brb3.70673, 40741677 PMC12311615

[4] Yu Y Zhang Y Zhu C Duan T Rao Z. Remnant cholesterol inflammatory index, calculated from residual cholesterol to C-reactive protein ratio, and stroke outcomes: a retrospective study using the national institutes of health stroke scale and modified Rankin scale. Lipids Health Dis. (2025) 24:228. doi: 10.1186/s12944-025-02650-2, 40604981 PMC12220354

[5] Han D Liu H Gao Y. The role of peripheral monocytes and macrophages in ischemic stroke. Neurol Sci. (2020) 41:3589–607. doi: 10.1007/s10072-020-04777-9, 33009963

[6] Manolis AA Manolis TA Melita H Mikhailidis DP Manolis AS. Low serum albumin: a neglected predictor in patients with cardiovascular disease. Eur J Intern Med. (2022) 102:24–39. doi: 10.1016/j.ejim.2022.05.004, 35537999

[7] Mehta A De Paola L Pana TA Carter B Soiza RL Kafri MW . The relationship between nutritional status at the time of stroke on adverse outcomes: a systematic review and meta-analysis of prospective cohort studies. Nutr Rev. (2022) 80:2275–87. doi: 10.1093/nutrit/nuac034, 35640017 PMC9647329

[8] Thuemmler RJ Pana TA Carter B Mahmood R Bettencourt-Silva JH Metcalf AK . Serum albumin and post-stroke outcomes: analysis of UK regional registry data, systematic review, and Meta-analysis. Nutrients. (2024) 16:1486. doi: 10.3390/nu16101486, 38794724 PMC11124370

[9] Zhou H Wang A Meng X Lin J Jiang Y Jing J . Low serum albumin levels predict poor outcome in patients with acute ischaemic stroke or transient ischaemic attack. Stroke Vasc Neurol. (2021) 6:458–66. doi: 10.1136/svn-2020-000676, 33632730 PMC8485231

[10] Deng C Liu B Wang M Zhu C Xu Y Li J . Analysis of the correlation between neutrophil percentage-to-albumin ratio, neutrophil-to-lymphocyte ratio and platelet-to-lymphocyte ratio with short-term prognosis in acute ischemic stroke patients undergoing intravenous thrombolysis. Front Neurol. (2025) 16:1512355. doi: 10.3389/fneur.2025.1512355, 40297858 PMC12034546

[11] Wang J Cao X Zeng S Zhou L Huang J Han Y . Nonlinear dose-response relationship between prognostic nutritional index and short-term outcome in acute ischemic stroke: a prospective cohort study. Front Nutr. (2025) 12:1529146. doi: 10.3389/fnut.2025.1529146, 40129670 PMC11930808

[12] Zhang ZL Guo QQ Tang JN Zhang JC Cheng MD Song FH . Monocyte-to-albumin ratio as a novel predictor of long-term adverse outcomes in patients after percutaneous coronary intervention. Biosci Rep. (2021) 41:BSR20210154. doi: 10.1042/bsr20210154, 34137842 PMC8243340

[13] Fu J Xu Y Chen X Li J Peng L. Monocyte-to-albumin ratio is associated with hematoma expansion in spontaneous intracerebral hemorrhage. Brain Behav. (2024) 14:e70059. doi: 10.1002/brb3.70059, 39344372 PMC11440023

[14] Yuan M Mao WE He X Zhang Q. Novel marker for predicting prognosis in hepatitis B virus-associated decompensated cirrhosis: monocyte-to-albumin ratio. Clin Lab. (2023) 69:2062–69. doi: 10.7754/Clin.Lab.2023.230204 37844048

[15] Dash UC Bhol NK Swain SK Samal RR Nayak PK Raina V . Oxidative stress and inflammation in the pathogenesis of neurological disorders: mechanisms and implications. Acta Pharm Sin B. (2025) 15:15–34. doi: 10.1016/j.apsb.2024.10.004, 40041912 PMC11873663

[16] Xie L He M Ying C Chu H. Mechanisms of inflammation after ischemic stroke in brain-peripheral crosstalk. Front Mol Neurosci. (2024) 17:1400808. doi: 10.3389/fnmol.2024.1400808, 38932932 PMC11199882

[17] Fang W Zhai X Han D Xiong X Wang T Zeng X . CCR2-dependent monocytes/macrophages exacerbate acute brain injury but promote functional recovery after ischemic stroke in mice. Theranostics. (2018) 8:3530–43. doi: 10.7150/thno.24475, 30026864 PMC6037034

[18] Weiss A Ding Y. Beyond reperfusion: adjunctive therapies targeting inflammation, edema, and blood-brain barrier dysfunction in ischemic stroke. Cerebrovasc Dis. (2025) 54:1–10. doi: 10.1159/000547092, 40609526 PMC12416540

[19] de Man AME Gunst J Reintam Blaser A. Nutrition in the intensive care unit: from the acute phase to beyond. Intensive Care Med. (2024) 50:1035–48. doi: 10.1007/s00134-024-07458-9, 38771368 PMC11245425

[20] Dal Bello S Ceccarelli L Tereshko Y Gigli GL D'Anna L Valente M . Prognostic impact of malnutrition evaluated via bioelectrical impedance vector analysis (BIVA) in acute ischemic stroke: findings from an inverse probability weighting analysis. Nutrients. (2025) 17:919. doi: 10.3390/nu17050919, 40077787 PMC11901430

[21] Jiang TT Zhu XY Yin YW Liu HJ Zhang GY. The prognostic significance of malnutrition in older adult patients with acute ischemic stroke. Front Nutr. (2025) 12:1529754. doi: 10.3389/fnut.2025.1529754, 39957766 PMC11825317

[22] Li D Liu Y Jia Y Yu J Li F Li H . Association between malnutrition and stroke-associated pneumonia in patients with ischemic stroke. BMC Neurol. (2023) 23:290. doi: 10.1186/s12883-023-03340-1, 37537542 PMC10399066

[23] Arques S. Human serum albumin in cardiovascular diseases. Eur J Intern Med. (2018) 52:8–12. doi: 10.1016/j.ejim.2018.04.014, 29680174

[24] Zhao J Chen M Mo J Zhong Y Qiu J Qiu Y . Prognostic value of albumin-based malnutritional indices on short-term outcome in acute ischemic stroke patients undergoing reperfusion therapy. Front Nutr. (2025) 12:1659446. doi: 10.3389/fnut.2025.1659446, 40832639 PMC12358433

[25] Della Corte V Tuttolomondo A Pecoraro R Di Raimondo D Vassallo V Pinto A. Inflammation, endothelial dysfunction and arterial stiffness as therapeutic targets in cardiovascular medicine. Curr Pharm Des. (2016) 22:4658–68. doi: 10.2174/1381612822666160510124801, 27160758

[26] Tuttolomondo A Petta S Casuccio A Maida C Corte VD Daidone M . Reactive hyperemia index (RHI) and cognitive performance indexes are associated with histologic markers of liver disease in subjects with non-alcoholic fatty liver disease (NAFLD): a case control study. Cardiovasc Diabetol. (2018) 17:28. doi: 10.1186/s12933-018-0670-7, 29452601 PMC5815178

[27] Tuttolomondo A Casuccio A Della Corte V Maida C Pecoraro R Di Raimondo D . Endothelial function and arterial stiffness indexes in subjects with acute ischemic stroke: relationship with TOAST subtype. Atherosclerosis. (2017) 256:94–9. doi: 10.1016/j.atherosclerosis.2016.10.044, 27817840

[28] Tuttolomondo A Di Raimondo D Pecoraro R Casuccio A Di Bona D Aiello A . HLA and killer cell immunoglobulin-like receptor (KIRs) genotyping in patients with acute ischemic stroke. J Neuroinflammation. (2019) 16:88. doi: 10.1186/s12974-019-1469-5, 30995924 PMC6471781

[29] Zanoli L Ozturk K Cappello M Inserra G Geraci G Tuttolomondo A . Inflammation and aortic pulse wave velocity: a multicenter longitudinal study in patients with inflammatory bowel disease. J Am Heart Assoc. (2019) 8:e010942. doi: 10.1161/JAHA.118.010942, 30712441 PMC6405571

[30] Kanazawa M Hatakeyama M. Intercellular communication between peripheral monocytes and central nervous system cells in stroke. Neural Regen Res. (2025) 21. doi: 10.4103/nrr.Nrr-d-25-00738, 41169215 PMC13568618

[31] Evans TW. Review article: albumin as a drug--biological effects of albumin unrelated to oncotic pressure. Aliment Pharmacol Ther. (2002) 16:6–11. doi: 10.1046/j.1365-2036.16.s5.2.x, 12423448

[32] Regitz-Zagrosek V Gebhard C. Gender medicine: effects of sex and gender on cardiovascular disease manifestation and outcomes. Nat Rev Cardiol. (2023) 20:236–47. doi: 10.1038/s41569-022-00797-4, 36316574 PMC9628527

[33] Sharma S Gibbons A Saphire EO. Sex differences in tissue-specific immunity and immunology. Science. (2025) 389:599–603. doi: 10.1126/science.adx4381, 40773572 PMC12777860

[34] Franceschi C Garagnani P Parini P Giuliani C Santoro A. Inflammaging: a new immune-metabolic viewpoint for age-related diseases. Nat Rev Endocrinol. (2018) 14:576–90. doi: 10.1038/s41574-018-0059-4, 30046148

[35] Klein SL Flanagan KL. Sex differences in immune responses. Nat Rev Immunol. (2016) 16:626–38. doi: 10.1038/nri.2016.90, 27546235

[36] Kong P Cui ZY Huang XF Zhang DD Guo RJ Han M. Inflammation and atherosclerosis: signaling pathways and therapeutic intervention. Signal Transduct Target Ther. (2022) 7:131. doi: 10.1038/s41392-022-00955-7, 35459215 PMC9033871

